# Deployment of biologging tags on free swimming large whales using uncrewed aerial systems

**DOI:** 10.1098/rsos.221376

**Published:** 2023-04-19

**Authors:** David N. Wiley, Christopher J. Zadra, Ari S. Friedlaender, Susan E. Parks, Alicia Pensarosa, Andy Rogan, K. Alex Shorter, Jorge Urbán, Iain Kerr

**Affiliations:** ^1^ Stellwagen Bank National Marine Sanctuary, National Oceanic and Atmospheric Administration, National Ocean Services, 175 Edward Foster Road, Scituate, MA 02066, USA; ^2^ Ocean Alliance, Inc., 32 Horton Street, Gloucester, MA 01930, USA; ^3^ Institute for Marine Sciences, University of California Santa Cruz, Santa Cruz 95064, CA, USA; ^4^ Department of Biology, Syracuse University, 114 Life Science Complex, Syracuse, NY 13244, USA; ^5^ Department of Mechanical Engineering, University of Michigan, 2350 Hayward, Ann Arbor, MI 48109, USA; ^6^ Department of the Coastal and Marine Sciences, Universidad Autónoma de Baja California Sur, La Paz 23084, Mexico

**Keywords:** uncrewed aerial systems, drones, whales, tagging, biologging

## Abstract

Suction-cup-attached biologging tags have led to major advances in our understanding of large whale behaviour. Getting close enough to a whale at sea to safely attach a tag is a major limiting factor when deploying these systems. Here we present an uncrewed aerial system (UAS)-based tagging technique for free-swimming large whales and provide data on efficacy from field testing on blue (*Balaenoptera musculus*) and fin (*B. physalus*) whales. Rapid transit speed and the bird's-eye view of the animal during UAS tagging contributed to the technique's success. During 8 days of field testing, we had 29 occasions when a focal animal was identified for attempted tagging and tags were successfully attached 21 times. The technique was efficient, with mean flight time of 2 min 45 s from launch to deployment and a mean distance of 490 m from the launch vessel to tagged animal, reducing potential adverse effects resulting from close approaches for tagging. These data indicate that UAS are capable of attaching biologging tags to free-swimming large whales quickly and from large distances, potentially increasing success rates, decreasing attempt times, and reducing animal disruption during tagging.

## 1. Introduction

Data acquired from biologging tags have become an important tool for studying free-swimming marine mammals around the world [1–3]. Data collected by these tags have provided important and novel information relative to behavioural ecology of large whales, often with the goal of mitigating anthropogenic interactions such as entanglement or ship strike [4,5] or reaction to noise [6]. To date, biologging tags have provided important information for many species of large whales, including humpback (*Megaptera novaeangliae*) [7–9], blue (*Balaenoptera musculus*) [10–12], sperm (*Physeter macrocephalus*) [13], sei (*B. borealis*) [14], North Atlantic right (*Eubalaena* *glacialis)* [4,15], fin (*B. physalus*) [16,17] and minke [18] whales. Data acquired from biologging tags also provide insights into swimming biomechanics [19,20], particular behaviours such as breaching mechanics [21] and ecosystem function [22].

In an effort to reduce the impact on the animal, suction cups have been used to attach several types of biologging tags to large whales, including: DTAGs [23], CATS tags [24] and Bioacoustic (B)-Probes [25,26]. Common to all these systems is a delivery method that uses a small boat to approach within metres of the whale, after which a pole is used to affix a tag to the animal (i.e. pole-tagging, see [7,8,27]). An alternative method has been developed using a pneumatic system to launch tags from up to 12 m using an aerial rocket tagging system [28]. These approaches have been successfully used to deploy tags on a variety of species (see above), and a number of organizations have developed considerable infrastructure and expertise for their use. However, considerable distances can occur between the tagging boat and surfacing animals, limiting tagging opportunities to distances that can be closed at safe operating speeds. This is particularly important for fast-moving species (e.g. fin and sei whales). In addition, the close approach required by these methods presents the possibility of harassment (e.g. physical disturbance and/or noise) and injury risk for whales and/or people. Here, we investigate the use of uncrewed aerial systems (UAS) as a platform for tagging free-swimming large whales.

The speed, manoeuvrability and bird's-eye view provided by a UAS are an advantage compared with traditional approach vessels. For example, many commercially available UAS can quickly reach in-air speeds in excess of 50 km h^−1^, meaning the tagging vessel can launch the UAS considerable distances from a surfacing animal and reach the whale to attempt tagging during that same surfacing. During an approach, the extreme manoeuvrability of the UAS and its constant birds-eye view of the animal means that, in favourable conditions, the pilot can track the focal whale when it is travelling subsurface between ventilations, thereby maintaining an optimal position for tagging when the animal surfaces. While there are many potential advantages of using UAS for tagging large whales, these systems have not been widely adopted for this purpose. The first published description of a UAS tag deployment describes a system for attaching tags on sperm whales (*P. macrocephalus*) [29].

In this paper, we present a UAS-based tag deployment system for motion-sensing and acoustic biologging tags, and the first successful use of a UAS to tag free-swimming baleen whales—blue and fin whales. Our findings demonstrate that UAS are capable of attaching biologging tags to free-swimming large whales with little observed behavioural response, and at greater stand-off distance and more quickly than traditional methods. We propose that the use of UAS for tagging activities will reduce (i) close-approach-related vessel disturbance, and (ii) time and number of approaches needed to attach a tag. Additionally, the boat with the UAS operator can be hundreds of metres from the whale, reducing potential for injury of both people and animals during a tagging approach.

## 2. Materials and methods

The tagging system presented in this work was designed to deploy commonly used suction-cup-equipped biologging tags (DTAGs [23] and CATS tags (Custom Animal Tracking Solutions, https://cats.is) [30]), rather than custom tags specifically designed and fabricated for use with a UAS (e.g. [29]). The CATS tag weighs approximately 560 g is 127 mm wide and 265 mm long. The DTAG is smaller, weighing approximately 200 g with a length of 148 mm and width of 82 mm. Two UAS, a DJI Matrice M210 V2 (dji.com/matrice-200-series-v2) and a DJI Inspire 2 (https://www.dji.com/inspire-2), were used to deploy the tags. The DJI Matrice M210 V2 was capable of flying with payloads greater than both DTAGs and CATS tags, and the DJI Inspire 2 was only used to carry DTAG payloads. The team has extensive experience operating these platforms. An iterative design–build–test approach, along with controlled experimental testing, was used to create the attachment system. Experimental testing was conducted to (i) quantify flight kinematics and kinetics at impact, (ii) examine how the UAS kinematics and propeller wash affects tag flight kinematics during the drop, and (iii) observe suction cup attachment to substrates during tagging simulations.

### 2.1. Tag drop dynamics

**Figure 1.**
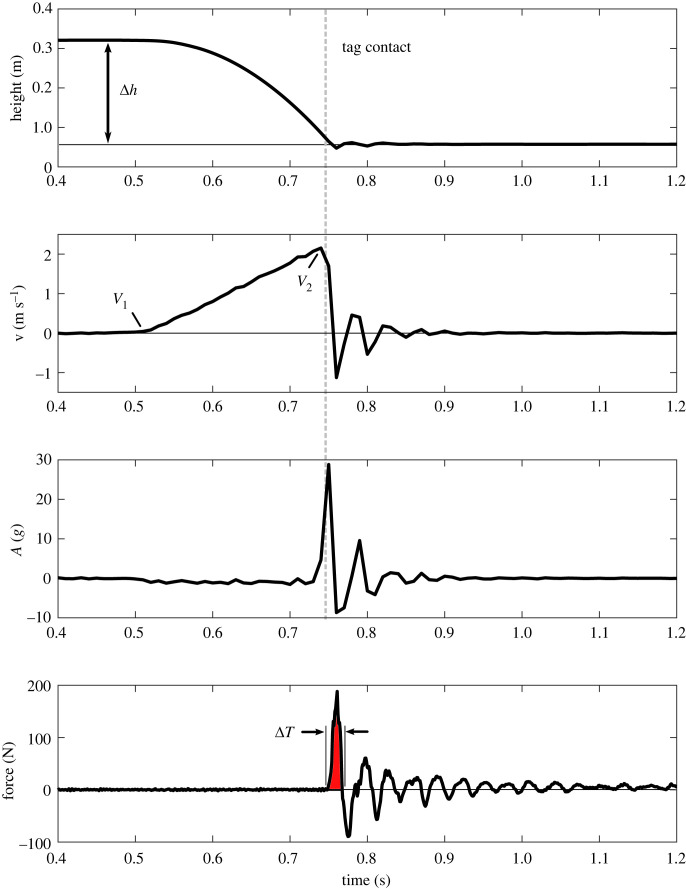
Representative kinematics and kinetics data from the drop testing in the laboratory. Measured position data were used to calculate speed and acceleration. Tag contact with the attachment surface is indicated by the dashed line.

To maximize our success during fieldwork, we conducted laboratory investigations into the fall behaviour of tags leading to attachment. Tag kinematics and the force at impact were measured experimentally to quantify the dynamics of the tagging system during a drop. Testing was conducted with a DTAG and the drop attachment system. A motion capture camera system (VICON, USA) sampled at 100 Hz, and force plate (Bertec, USA) sampled at 1000 Hz were used to collect kinematic and kinetic data. Speed and acceleration of the tag system during the drop were calculated numerically from the position data. The combined system had a mass of 0.48 kg and was dropped indoors by hand from heights of 0.3, 1.3 and 2.2 m (figure 1). The attachment surface consisted of a 0.6 cm acrylic plate with a 2.5 cm silicone interface between the acrylic and force plate. Drops were repeated three times at each height. Preliminary testing indicated that a drop height of 1.8 m was required to create an impact force that would fully compress the cups on the attachment surface. The change in linear moment was used to estimate the force at attachment and was verified using experimental measurements.

2.1 $$mv_{2}-mv_{1}=\overset{t2}{\underset{t1}{\int }}F(t)dt,$$

where *m* is the mass of the combined system, *v* is the speed during flight and *F* is the impulsive force at impact. To estimate the speed of the system just before contact during the field deployments, we assumed that the aerodynamic forces acting on the tag could be simplified to the following relationship:

2.2 $$\hat{F_{d}}(t)\;=\frac{1}{2}\rho AC_{d}\;v^{2}(t),$$

where the drag force (*F_d_*) during the drop is a function of the speed (*v*), mass density of the fluid (ρ), the cross-sectional area of the tag (*A*) and the drag coefficient $\left(C_{d}\right)$. The drag coefficient was estimated using the experimental drop data. The simplified drag force and the gravitational force are then related to the system acceleration using

2.3 $$a(t)=\frac{1}{2m}\rho AC_{d}\;v^{2}(t)-g.$$

This differential equation was then solved numerically to estimate the position and speed of the system using the ODE solver ODE45 in Matlab (Natick, MA, USA). Simulations were run for each successful DTAG deployment to estimate the speed of the combined system just before impact. These speeds were used with (2.1) to estimate average impact force.

### 2.2. Uncrewed aerial system drop testing

In addition to the testing used to quantify flight kinematics and impact kinetics, we conducted 179 land-based testing trials using the DJI Matrice M210 V2 and the DJI Inspire 2 to drop tags in flight. The DJI Matrice M210 V2 was the primary UAS system for the testing and fieldwork. The smaller DJI Inspire 2 could only safely carry the lighter DTAG and was used as a backup system for DTAG deployments. Data were collected using high-speed cameras (GoPro Hero 8, iPhone 11, Sony Cyber-Shot RX100 VII), and tags were dropped from heights ranging from 1 to 6 m. Qualitative analysis from video taken during these tests was used to better understand how external environmental disturbances (propeller wash from the UAS, UAS flight dynamics, wind) affected the flight stability of the tags during the drop. During the testing, six 12 × 14 × 3 inch blocks of ballistic gel (clearballistics.com/) were used as a proxy for the whale. To simulate a field deployment, we dropped tags on both wet and dry surfaces, and with the substrate at a range of angles with respect to the horizontal (electronic supplementary material, video S1). Further, we used moving targets to evaluate tag attachments and for pilot training. We accomplished this by attaching ballistic gel to various moving platforms both on land with a hand-pulled cart and on the water using a small dinghy to tow a gel-equipped surfboard at speeds between 5 and 12 km h^−1^. A GoPro Hero 8 (gopro.com) was attached to film tag drops and interactions with ballistic gel in slow motion, and the camera on the UAS recorded for the duration of each flight (electronic supplementary material, video S2). We considered a tagging attempt successful if one or more suction cups adhered to the surface.

### 2.3. Drop system

**Figure 2.**
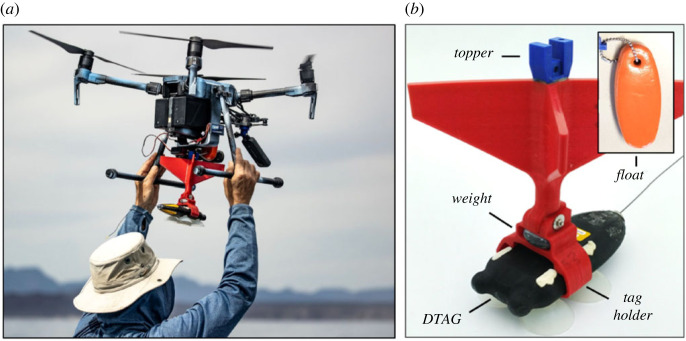
(*a*) Hand launching assembled M210 V2 with the DTAG deployment package. (*b*) DTAG drop system components.

**Figure 3.**
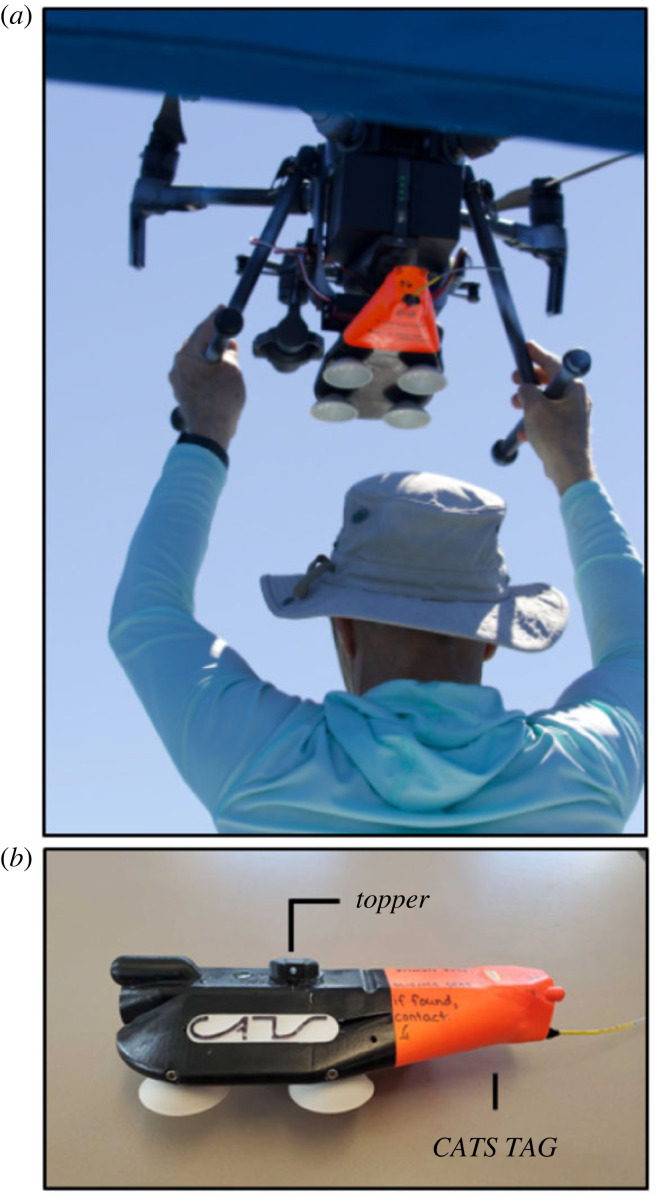
(*a*) M210 V2 with a CATS tag. *(b)* The ‘topper’ that was added to the CATS tag to interface with the pin-based release system.

**Figure 4.**
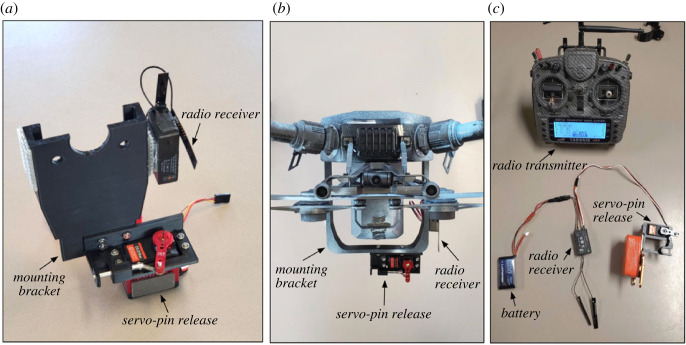
(*a*) DJI Inspire 2 mounting bracket and servo-pin release. (*b*) DJI M210 mounting bracket and servo-pin release. (*c*) Release system components—radio transmitter, radio receiver, battery and servo-pin release.

**Figure 5.**
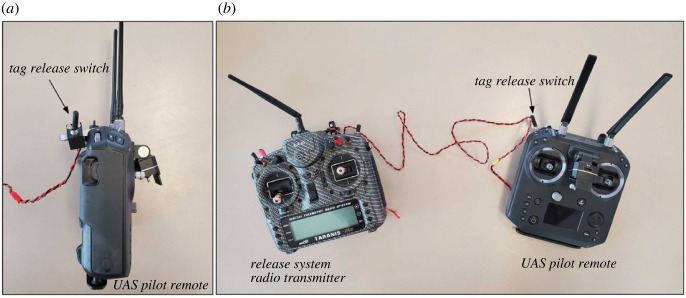
(*a*) Side view of additional switch mounted to UAS pilot remote. (*b*) Additional switch mounted to UAS pilot remote connected via extended wire to the release system radio transmitter for single-operator control of UAS and release system.

In order for the tag to attach successfully, the suction cups must be oriented parallel to the animal at the time of impact. However, the aerodynamics and flight stability of this tag orientation are poor, particularly for the smaller and lighter DTAG. To compensate, we fabricated a secondary attachment system (the drop system) to improve the flight stability of the tag. This system consisted of (i) a releasable interface (the tag holder) that connected the tag to the drop system, (ii) a shaft with four fins to generate aerodynamic forces to improve stability, and (iii) the connection to the release system on the UAS (the topper). The topper was designed to rigidly attach to the release system to prevent unwanted motion of the drop system during flight. All of the components were three-dimensional printed using TPU and PLA plastics. The force at impact was large enough to separate the tag from the drop system, enabling recovery and reuse. A 115 g mass was added to the tag holder to further separate the centre of mass from the fins and increase the average impact force at contact. The final assembly weighed 215 g, and foam flotation was included to ensure that the combined system was positively buoyant (figure 2). The flight dynamics of the heavier CATS tags were more stable, and this tag was dropped without a secondary attachment system. CATS tags were connected directly to the UAS with a lower profile topper (figure 3) that was glued onto the tag over the centre of mass. Both tagging systems were released from the UAS using a remote-controlled servo-pin release system consisting of the following components: (i) mounting brackets designed for each system were used to attach the release system to the UAS, (ii) a servo-pin release (servo, servo bracket and pin) was fixed to the mounting bracket, (iii) a 2.4 GHz radio receiver (FrSky X8R) and battery to power and control the servo, and (iv) a 2.4 GHz radio transmitter (Taranis X9D Plus) with a switch programmed to open and close the servo (figure 4). The switch from the Taranis X9D used to activate the release system was mounted to the UAS pilot remote via an extended wire to allow for a single operator to both pilot the UAS and activate the release system (figure 5). All CAD files are provided in the electronic supplementary material.

### 2.4. Field data collection

Tagging was conducted from 23 February to 9 March 2022, in the waters around Loreto Bay National Park, Mexico (26°0′42.32″ N, 111°20′51.91″ W). We focused on blue and fin whales using two CATS tags and four DTAGs. The objective of the field effort was to demonstrate the viability of the UAS attachment method. As such, the tags were set to release after 1–3 h, allowing same-day redeployments to maximize the number of unique tag deployments. Additionally, four ‘expendable tags’ (i.e. without sensors or electronic function) with the same size and weight of a DTAG were also used to tag the animals. This approach increased the number of overall tag attachment attempts, even though they did not collect data from the animals.

**Figure 6.**
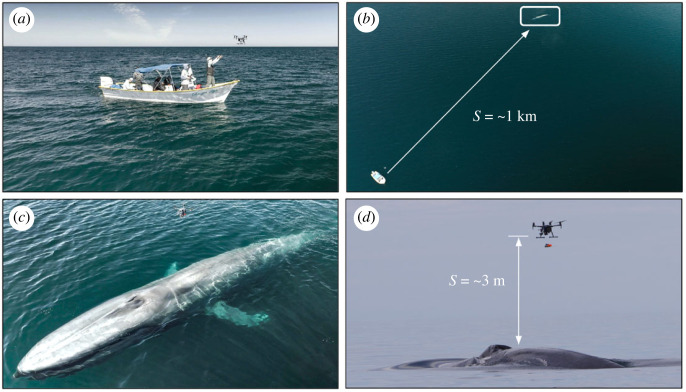
(*a*) Hand launch and recovery of UAS from boat. (*b*) Animals tagged at distances of over 1 km without the need to rapid/close approach. (*c*) UAS with CATS tag payload over blue whale. (*d*) A representative tagging attempt with a CATS tag from approximately 3 m.

Research was conducted from an 8 m panga-style boat. After identifying an animal for a tagging attempt, the UAS was prepared and launched as soon as the animal surfaced after an extended dive (figure 6). The UAS pilot operated the aircraft by remote control with a mounted tablet (DJI CrystalSky Ultrabright) and monitored animal activity through the aircraft's onboard gimbal-mounted 4 K first-person view camera, transmitted to a live feed on the tablet. We recorded video for the entire duration of the UAS flight. After the UAS was launched, the pilot gained altitude and relocated the identified animal. The pilot transited to the identified animal at a speed of up to 50 km h^−1^. Approaching the animal from behind, the pilot reduced speed to match the speed of the animal and positioned the UAS over the whale for the tag attempt at an altitude of approximately 7.4 m for DTAGs and 3.5 m for CATS tags. When in position over the whale, the pilot changed flight modes to *Attitude Mode* (ATTI-mode) and adjusted the camera to 90 degrees down so that the whale was centred in the tablet screen. In ATTI-mode, the UAS automatically maintains altitude but not its GPS location. The benefit of this flight mode is that the UAS maintains its vector heading when the pilot releases the stick controls, while also maintaining its altitude and horizontal orientation (i.e. 0 degrees pitch and roll) over the animal. This allows the tag to have a horizontal orientation at the time of release (electronic supplementary material, video S3). Diagonal grid lines were used to identify the centre point on the screen and as a target when the tag was released from the UAS. The pilot was careful not to fly over the head of the animal, using the pectoral fins as a guide for positioning the UAS safely over the back of the animal. Occasionally, a second smaller UAS (DJI Mavic 3 cine) was flown to film the tagging sequence and monitor changes in whale behaviour (electronic supplementary material, video S4).

A combination of direct observations and data automatically collected and archived by the UAS in its flight logs were used to determine the efficacy of the UAS as a tagging platform. UAS flight logs were analysed using *AirData* analysis software (airdata.com). For each flight, we collected the following: date, flight number, tag type and unique identifier, tag-holder type, approach number, whale species and the number of whales in the focal group. We used direct observation or UAS camera footage to record if and when a tag was released, if the tag made contact with the whale and if attachment was successful. *AirData* was combined with direct and/or video observations to record UAS launch time, launch location (latitude and longitude), time of first contact with the whale (defined as the UAS positioned over the animals and appearing in the pilot's view screen), time from launch to first contact, distance from launch to first contact, time of tag release, altitude at tag release, horizontal speed at time of release, time from first contact to tag release and distance from launch to tag release. A tag attempt occurred when the tag was released from the UAS towards the whale. A tagging attempt was considered successful if the pilot observed adhesion of one or more suction cups to the whale and the tag remained adhered during the animal's subsequent dive. We used direct observation and video data from the UAS to judge the reaction of the whales to the tagging event based on a 1–4 scale, with ‘1’ being no observed reaction and ‘4’ being a strong reaction (i.e. high energy behaviour such as tail slashes or speed swimming) [31].

## 3. Results

### 3.1. Tag drop dynamics

**Figure 7.**
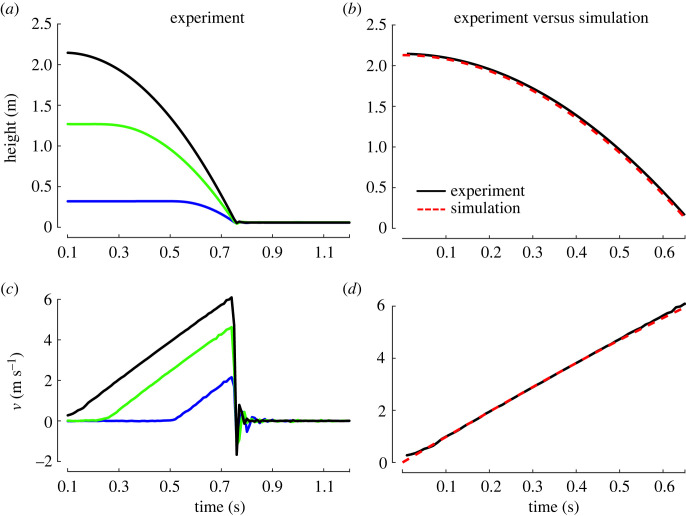
(*a*,*c*) Representative kinematic data from the three experimental drop heights in the laboratory. (*b,d*) Simulated and experimental drop height and speed compare well.

**Figure 8.**
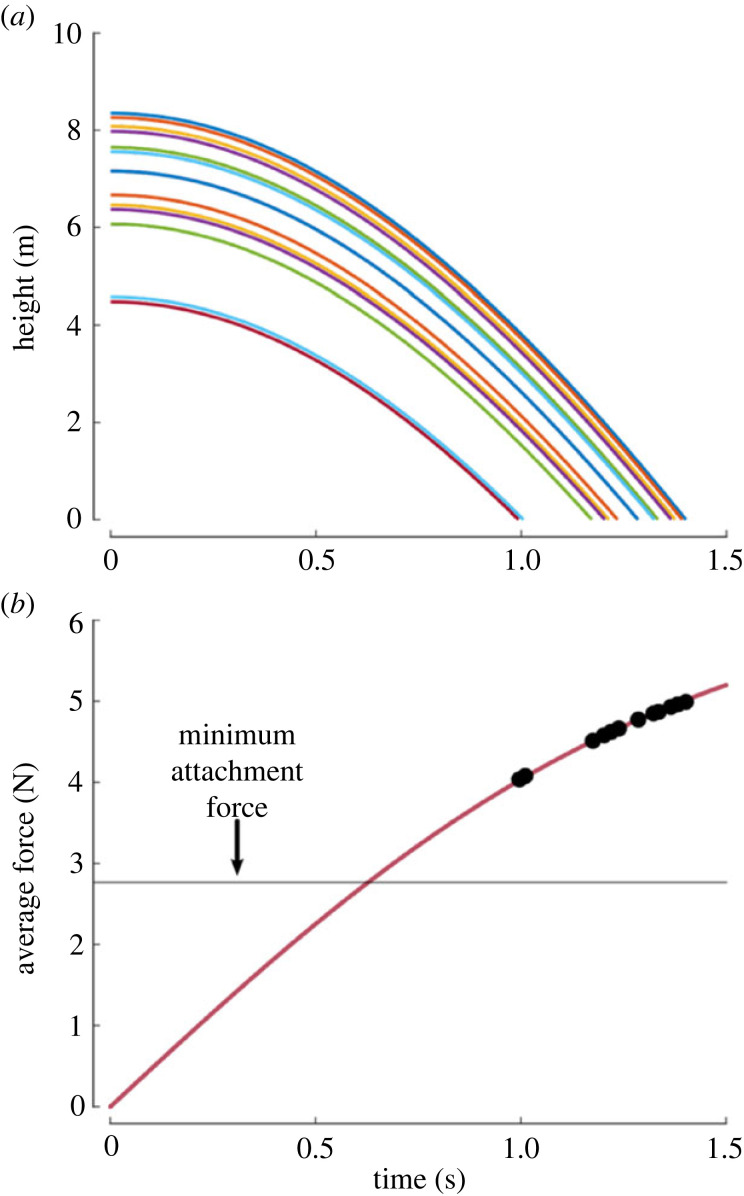
(*a*) Simulations of the drops for each of the successful DTAG drops. (*b*) Estimates of the average impact force for each of the drops. All of the deployments had estimated forces large enough to full compress the cups.

During the in-laboratory testing, tag speed at impact ranged from approximately 2 to 6 m s^−1^, generating an average impact force of approximately 1 N at the lowest drop and approximately 3 N when dropped from slightly more than 2 m. Experimental data were used to heuristically identify the drag coefficient (Cd = 3.15) for the combined system. Figure 7 shows good agreement between experimental data and simulation results for a 2.1 m drop in the laboratory. Tag drops for each of the successful deployments were then simulated and used to estimate the average impact force, figure 8. The drop height averaged 6.9 ± 1.2 m and ranged from 4.5 to 8.4 m. The change in linear momentum at impact was used to estimate an average impact force of 4.6 ± 0.3 N for all the successful drops. The average impact force ranged from 4 to 5 N.

### 3.2. Uncrewed aerial system drop testing

Results from the drop testing indicated that there were several factors that influenced the fall behaviour of the tag and its ability to attach: (i) the movement of the UAS at the time of tag release, (ii) the design of the mechanism that secured and then released the tag from the UAS (the ‘release system’), and (iii) the tag characteristics that facilitated or impeded its ability to land suction cup down (the drop system). Excessive movement of the UAS at the time of tag release resulted in unpredictable initial pitch and roll and poor orientation stability during the drop. This movement was mitigated by placing the UAS in *Attitude Mode* (ATTI-mode) when manoeuvring the UAS before tag release. The design of a custom ‘topper’ to interface with the servo-pin release system was key to a clean release from the UAS (figures 2 and 3). The toppers were designed to rigidly attach to the release mechanism with very tight tolerances to reduce tag movement while in flight. Therefore, the pitch and roll orientation of the tag was directly related to the pitch and roll of the UAS, which are controlled by the pilot. Rigid attachment close to the centre of mass of the UAS maintained flight stability without having to adjust or modify flight controller settings on the UAS. Observations of the drop kinematics from the testing were used to inform the design of the DTAG drop system (see below).

### 3.3. Drop system

**Figure 9.**
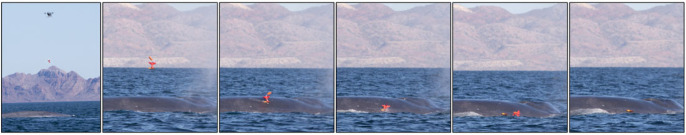
Photo sequence showing DTAG deployment using the drop system, and the release of tag holder and fins on impact that float for recovery and reuse.

The finned and weighted configuration of the DTAG drop system improved flight stability during the drop. Drop heights greater than approximately 6 m provided enough time for the system to orient the tag suction cups down at impact. The added foam flotation also acted as a drogue trailing behind the falling tag and helped to orient the system into the suction-cup-down position. The resulting impact force secured the cups to the surface and detached the fins from the DTAG for retrieval and reuse (figure 9; electronic supplementary material, video S5). CATS tags could be dropped without fins or additional weight. The centre of mass was unique to each tag and the location of the ‘topper’ was different for each tag. Test drops during level flight with minimal horizontal movement demonstrated that drop heights of 3–4 m generated sufficient force to secure the cups to the surface. The CATS tags tended to maintain a stable cups-down orientation during drops from this height, but if dropped from greater height, or during left or right manoeuvring of the UAS, tags lost stability and tumbled in the air.

### 3.4. Field results

We identified and attempted to tag focal animals on 29 separate occasions, resulting in 21 successful tag attachments, a success rate of 72% (table 1). These 21 successful attachments required 57 total UAS flights, primarily using the DJI Matrice M210 V2 (95%, *n* = 54). On average, each focal animal was approached with the UAS twice, and 1.6 tag drops were made during a tagging attempt. In total, 86% (49/57) of the flights resulted in the UAS successfully stationed above the focal animal (i.e. the UAS was suitably positioned over the whale for a tag drop). Of these, 73% (36/49) resulted in a tag drop (i.e. the tag was released from the UAS towards the animal). Of the tag drops, 72% (26/36) made contact with the whale, with 81% (21/26) of the tags that made contact resulting in attachment. Some flights were terminated prior to the drone being stationed above an animal or attempting to tag, due to environmental or other outside factors.

**Table 1.** Summary of UAS tagging activities directed at blue and fin whales showing the number of (i) days in the field, (ii) occasions where a focal animal was identified for a tag attempt, (iii) UAS flights, (iv) tag drops (releasing the tag from the UAS towards the whale), and (v) successful tag attachments.

day	focal animal tagging attempt	number of flights	number of drops	tag attachment success
23 Feb	1	4	3	yes
	2	2	2	yes
	3	2	2	yes
	4	5	1	yes
28 Feb	5	1	1	yes
	6	1	0	no
1 Mar	7	1	1	yes
	8	5	2	no
	9	1	1	yes
2 Mar	10	3	2	yes
	11	1	1	yes
	12	1	1	yes
	13	3	1	no
	14	2	2	no
3 Mar	15	1	1	yes
	16	2	1	yes
	17	3	2	yes
	18	2	1	no
4 Mar	19	1	1	no
	20	1	1	yes
5 Mar	21	1	1	yes
	22	3	1	no
	23	1	1	yes
	24	1	1	yes
9 Mar	25	1	1	yes
	26	1	1	yes
	27	3	2	yes
	28	1	0	no
	29	3	1	yes
	29 focal animal tagging attempts	57 flights	36 drops	21 successful attachments

#### 3.4.1. Tag attachment by species

Fifty flights were directed at blue whales. In 86% (43/50) of the flights, the UAS successfully stationed above the focal whale. Of these, 72% (31/43) resulted in a tag drop. Of the dropped tags, 68% made contact with the animal (21/31) and 52% (16/31) of the dropped tags resulted in successful tag attachment (table 2). Seven flights were directed at fin whales. In 86% (6/7) of the fights, the UAS successfully stationed above the focal whale. Of these, 83% (5/6) resulted in a dropped tag. Of the dropped tags, 100% made contact with the animal, and 100% of the dropped tags resulted in successful tag attachment (table 2).

**Table 2.** Summary of UAS tagging activities directed at blue and fin whales showing the number of (i) UAS flights, (ii) times the UAS was stationed above the whale and in a position to tag the animal, (iii) per cent of flights resulting in UAS stationed above the animal, (iv) tag drops (defined as releasing the tag from the UAS towards the whale), (v) per cent of UAS stationings that resulted in a tag drop, (vi) tag drops that resulted in the tag landing on the whale, (vii) per cent of tag drops that resulted in a landed tag, (viii) landed tags that successfully attached to the whale, and (ix) per cent of landings that resulted in successful attachment.

species	flights	UAS stationed above whale	% UAS stationed above whale out of flights	tag drops	% tag drops out of UAS stationed above whale	tag landed on whale	% successful landings out of tag drops	successful attachments	% successful attachments out of tags landed on whale
blue	50	43	86% (43/50)	31	72% (31/43)	21	68% (21/31)	16	76% (16/21)
fin	7	6	86% (6/7)	5	83% (5/6)	5	100%	5	100%
total	57	49	86% (49/57)	36	73% (36/49)	26	72% (26/36)	21	81% (21/26)

#### 3.4.2. Comparing DTAGs and CATS tags

We made 42 flights using DTAGs and 15 using CATS tags. Of the 15 CATS tag flights, 10 resulted in a dropped tag. Of these, 80% (8/10) made contact with the whale, with 63% (5/8) of the tags that made contact successfully attaching. Thus, 50% (5/10) of tag drops resulted in a CATS tag attached to a whale (table 2). The mean height at tag drop was 3.5 m (s.d. = 0.8 m, range = 2.6–3.9 m). Mean UAS speed at the time of drop was 5.7 km h^−1^ (s.d. = 2.8 km h^−1^, range = 1.9–7.8 km h^−1^) (table 3).

**Table 3.** Summary of UAS tagging activities directed at blue and fin whales, showing the number of (i) tag drops (defined as release the tag from the UAS towards the whale), (ii) tag drops that resulted in the tag landing on the whale, (iii) per cent of tag drops that resulted in a landed tag, (iv) landed tags that attached to the whale, (v) per cent of landings that resulted in attachment, (vi) per cent of tag drops that resulted in attachment and summary of (vii) UAS altitude, and (viii) speed from all tag drops by each tag type (*n* = 36, CATS *n* = 10 DTAG *n* = 26).

tag type	tag drops	tags landed on whale	% tags landed out of drops	successful attachments	% tags landed resulting in attachment	% drops resulting in attachment	UAS altitude at drop (m)		UAS speed at drop (km h^−1^)	
							mean	s.d.	mean	s.d.
CATS	10	8	80% (8/10)	5	63% (5/8)	50% (5/10)	3.5 (range 2.6–3.9)	0.8	5.7 (range 1.9–7.8)	2.8
DTAG	26	18	69% (18/26)	16	89% (16/18)	62% (16/26)	7.4 (range 4.5–8.4)	1.5	6.4 (range 4.0–10.0)	2.7
total	36	26	72% (26/36)	21	81% (21/26)	58% (21/36)				

We made 26 tag drops with DTAGs. Of these, 69% (18/26) made contact with the whale, with 89% (16/18) of the tags that made contact successfully attaching. Thus, 62% (16/26) of dropped tags resulted in a DTAG attached to a whale. The mean height of the UAS at the time of tag drop was 7.4 m (s.d. = 1.5 m, range = 4.5–8.4 m). Mean UAS speed at the time of drop was 6.4 km h^−1^ (s.d. = 2.7 km h^−1^, range = 4.0–10.0 km hr^−1^) (table 3).

#### 3.4.3. Distance and time from launch to tag drop

The mean distance from launch location to tag drop location (i.e. location where the tag was released from the UAS and dropped towards the whale) was 490.1 m (s.d. = 235.8 m, range = 90.1–1048.8 m). The mean time from launch to tag drop was 2 min, 45 s (s.d. = 2 min, 15 s; range 00 min, 43 s–12 min, 20 s).

#### 3.4.4. Animal reaction to tagging

Of the 49 flights in which the UAS successfully stationed above the focal whale, 10 resulted in No reaction, 34 resulted in a level 1 reaction—minor response (unsolicited dive or shallow dive), and five resulted in a level 2—moderate response (rapid dive).

## 4. Discussion

UAS-based tagging has the potential to significantly enhance the study of cetaceans in the wild. Our promising initial results demonstrate, to the best of our knowledge, the first substantive investigation into the use of UAS for attaching broadly used biologging tags (DTAGs and CATS) to free-swimming baleen whales (blue and fin). There is one published account of using a UAS to attempt attachment of a specially designed tag to sperm whales [29]. Murakami *et al*. [29] conducted 38 flights resulting in two partial attachments out of 11 tag drops. In each of the two attachments, the tag became detached prior to the animal diving, which would not have met our definition of a successful attachment (i.e. the tag did not remain attached during the animal's subsequent dive). During 8 days of field testing, we identified and attempted to tag individuals on 29 separate occasions, resulting in 21 successful tag attachments, a success rate of 72%. These 21 successful attachments required 57 total UAS flights, of which the tag was dropped 36 times (i.e. released from the UAS towards the whale). On average, each focal animal was approached twice, and 1.6 tag drops were made during a focal animal tagging attempt. These data, and the UAS-based tagging methodology presented in this work, clearly demonstrate the viability of this method to effectively attach tags to targeted individuals, while lessening the potential impact to the animal, including reducing the number of permitting takes used during the deployment.

During the fieldwork, we were able to evaluate the viability and efficacy of UAS-based tag deployments. A deployment was considered successful if the pilot or researcher observed suction cup attachment at impact, and the pilot could verify the tag remained secured during the animal's subsequent dive. The pilot used their own discretion on when to release the tag from the UAS and did not drop the tag unless they felt there was a high probability of success. The rapid transit speed and view perspective of the UAS allowed the pilot to track the animal subsurface between ventilations and remain in tagging position for the next surfacing. These advantages might also provide the ability for greater specificity during tagging operations when deciding which individuals to target. For example, on day 8 of our field operations, we identified a pair of travelling fin whales and were able to tag both individuals within 17 min, requiring only one UAS flight per individual. The lack of disturbance and ease of identifying already-tagged individuals is extremely beneficial when attempting to tag numerous animals in a behavioural group, which is a goal of many investigations.

Of the 21 deployments, 15 recorded data and the remaining six were deployments using the expendable tags. Tags were configured to release from the animal after only short periods of attachment (typically 0.5–1.5 h for DTAGs and 2–3 h for CATS tags). The short release times provided time to collect limited post-tagging behavioural data, while maximizing our ability to recover and redeploy tags on the same day, thereby increasing our sample size for attachment attempts. We also used seven expendable DTAGs that did not have to be retrieved, thereby increasing the number of overall tag attachment attempts we had available. One tag that did not release as designed, collected data on the animal until the tag stopped recording after 13 h, indicating that multiple-hour attachments using a UAS are possible, although additional research relative to attachment duration and comparison with other tagging methods is recommended.

Our data suggest that the reaction of blue and fin whales to the use of UAS for tagging was minimal, which is consistent with past investigations of baleen whales and reactions to UAS (see review in [2]). However, we recommend that a directed study of baleen whale reaction to UAS specific to tagging activities be conducted, particularly since our success could result in widespread use of the technique. Such studies could involve tag data being analysed to identify shallow and deep dives for foraging behaviours in each deployment using features in the depth and orientation data. Direct observation of feeding behaviours on dives subsequent to the tag event could also be used in the case of CATS or other video recording tags.

In this work, we used a design–build–test approach to iterate on the tagging system used to successfully attach two tag types (DTAG and CATS). Land trials were conducted to investigate UAS flight dynamics with the tags and tag drop dynamics after release. We used insights from these trials to develop tag-specific drop systems and protocols that increased the probability of the tag contacting in the suction-cup-down orientation needed for attachment, but the key parameters for successful attachment were the same: (i) Sufficient speed at contact to generate an attachment force large enough to fully deform the suction cups, and (ii) a cups-down tag orientation at contact. Physical parameters of the tags are directly related to performance during a drop, and the two tag types varied in weight and surface area. Additionally, aerodynamic forces imparted to the tags by the UAS propeller wash further complicated the drop dynamics.

Drop dynamics of the DTAG system were measured in the laboratory and used to identify parameters for a model to estimate average attachment force for DTAGs deployed in the field. The suction cups used with the DTAGs require approximately 2.8 N of force for full compression. The combined system weighs 0.48 kg and needs to reach a drop speed of at least 6 m s^−1^ to create a sufficient force. For a system released from rest this requires a drop height of at least 2 m. In the field, the DTAG system was dropped from an average height of 6.9 m, more than three times the minimum height, to give the drop system time to reorient to a cups-down orientation at contact. The larger initial drop heights resulted in an average estimated contact force of 4.7 N, 68% larger than required. The higher DTAG drops, however, resulted in more drops that missed the whale compared with the lower CATS tag drops. The added drop height increased the drop time to the animal and decreased the size of the target area visible from the pilot's point of view, resulting in more misses when the animal or the UAS changed direction during the drop. The specially designed drop system, however, resulted in a reliable suction-cup-down orientation, with speeds sufficient for cup attachment, at contact. While the lower CATS tag drops were more likely to make contact with the animal, unstable drop dynamics often resulted in poor cup orientation at contact. The development of a CATS drop system to correct orientation during the drop would probably improve performance.

To further improve drop accuracy, we developed a single-operator system, whereby the UAS pilot both controlled the UAS and released the tag. The release mechanism was activated using a separate remote-control system (Taranis X9D) than the UAS. However, the switch from the Taranis X9D used to activate the release system was mounted on the UAS pilot remote via an extended wire. This single-operator set-up was important to eliminate any lag time due to communication between the pilot and a dedicated person for releasing the tag. This also reduced the chance of release timing errors due to miscommunication. These mechanisms allowed a tag release at the time and orientation consistent with what the UAS pilot expected.

In summary, our results support the use of UAS as a platform for tagging free-swimming large whales. The UAS was able to successfully attach tags on specified individuals and do so from large initial distances in a short period of time. This potentially increases tagging efficiency, while reducing possible disturbance related to the close approach and required proximity typically needed to attach tags using small vessels. The technique also reduces the possibility of whales or people being struck and injured. Further developments could lead to UAS-based tagging methods becoming a viable method for vulnerable or hard-to-study species. However, it is important to emphasize the skill required of the UAS pilot, including extensive pre-flight training specific to tagging whales. Special permission or waivers may also be required in some countries to drop objects from UAS.

## Ethics

The fieldwork was conducted under Mexico permit SGPA/DVGS/10021/2.

## Data Availability

The datasets supporting this article have been uploaded as part of the electronic supplementary material [32].
